# Lack of interleukin-1 type 1 receptor enhances the accumulation of mutant huntingtin in the striatum and exacerbates the neurological phenotypes of Huntington's disease mice

**DOI:** 10.1186/1756-6606-3-33

**Published:** 2010-11-02

**Authors:** Chuan-En Wang, Shihua Li, Xiao-Jiang Li

**Affiliations:** 1 Department of Human Genetics, Emory University, Atlanta, GA 30322, USA

## Abstract

Huntington's disease results from expansion of a glutamine repeat (>36 glutamines) in the N-terminal region of huntingtin (htt) and is characterized by preferential neurodegeneration in the striatum of the brain. N171-82Q mice that express N-terminal 171 amino acids of htt with an 82-glutamine repeat show severe neurological phenotypes and die early, suggesting that N-terminal mutant htt is pathogenic. In addition, various cellular factors and genetic modifiers are found to modulate the cytotoxicity of mutant htt. Understanding the contribution of these factors to HD pathogenesis will help identify therapeutics for this disease. To investigate the role of interleukin type 1 (IL-1), a cytokine that has been implicated in various neurological diseases, in HD neurological symptoms, we crossed N171-82Q mice to type I IL-1 receptor (IL-1RI) knockout mice. Mice lacking IL-1RI and expressing N171-82Q show more severe neurological symptoms than N171-82Q or IL-1RI knockout mice, suggesting that lack of IL-1RI can promote the neuronal toxicity of mutant htt. Lack of IL-1RI also increases the accumulation of transgenic mutant htt in the striatum in N171-82Q mice. Since IL-1RI signaling mediates both toxic and protective effects on neurons, its basal function and protective effects may be important for preventing the neuropathology seen in HD.

## Background

Huntington's disease is characterized by late-onset neurodegeneration that occurs preferentially in the striatum [1,2]. This selective neurodegeneration is caused by expansion of a polyglutamine (polyQ) tract in the N-terminal region of huntingtin (htt), a large protein that consists of 3144 amino acids and is expressed ubiquitously in all types of cells throughout the body and brain. N-terminal fragments of mutant htt can affect intracellular trafficking [3-6] and enter the nucleus to alter gene expression [7,8]. Although we know that an expanded polyQ causes the misfolding of N-terminal htt and induces cytotoxicity, the mechanism underlying the selective neurodegeneration remains unclear.

Transgenic mouse models expressing mutant htt make a highly valuable model for untangling the pathogenesis of HD. Of a number of HD mouse models that have been established, N171-82 HD mice have been investigated extensively because of their robust neurological phenotypes. N171-82Q mice express N-terminal 171 amino acids of human htt with an 82Q repeat under the control of the prion promoter, which drives gene expression at a high level in neuronal cells [9]. As a result, mutant htt is abundantly expressed in neuronal cells in the cortex and striatum of N171-82Q mice, causing motor function deficits, body weight loss, and early death that often occurs at 5-6 months in these mice [9]. The robust and progressive neurological phenotypes of N171-82Q mice offer an advantage for identifying therapeutics that can alleviate neurological symptoms and for uncovering pathogenic mechanisms [10,11]. Nonetheless, although the involvement of neuroinflammation and cytokines are known to be involved in a number of neurological disorders, their roles in HD remain elusive.

Interleukin-1 (IL-1), a 17-kDa polypeptide, is the prototypical cytokine with pleiotropic effects and is dramatically upregulated during neuroinflammation [12]. Considerable efforts have been made to alter IL-1 signaling for the purpose of reducing the inflammation in acute pathological conditions. However, the effects of IL-1 appear to differ depending on its expression levels and the target organ. IL-1 functions as a mediator or inhibitor of diverse forms of neurodegeneration [12,13]. For example, IL-1 activates NF-kB signaling [12,14], which can be either protective of or destructive to cells [14,16]. Mutant htt can reduce NF-kB [17] and also increase NF-kB signaling [18] in cultured cells. Given both the pathogenic and protective functions of IL-1 and NF-kB, it is important that we understand the contribution of IL-1 signaling to the pathogenesis of HD.

Two well-characterized isoforms of IL-1, IL-1alpha and IL-1beta [19], can bind type I IL-1 receptor (IL-1RI) to trigger IL-1 signaling pathways. An endogenous antagonist (IL-1Ra) binds to IL-1RI and blocks IL-1 binding and signaling [20], conferring protection against pathologic events caused by the elevated level of IL-1. In this study, we crossed N171-82Q HD mice to IL-1RI knockout mice, in which NF-κB activation is inactive at all times [12,14]. We found that diminishing IL-1RI could increase the accumulation of mutant htt in the striatum and exacerbate the neurological symptoms of N171-82Q mice. Our findings suggest that the basal activity of IL-1RI is important for preventing the accumulation of mutant htt in striatal neurons and hence related neuropathogenic events, which could be helpful for the development of new strategies to treat HD.

## Methods

### Animals

All animal procedures were approved by the Institutional Animal Care and Use Committee of Emory University. N171-82Q mice (B6C3F1/J-Tg(HD82Gln)81Dbo/J, *Jackson Laboratory*, Bar Harbour, ME, USA) and IL-1RI knockout mice (B6.129S7-*Il1r1*^*tm1Imx*^/J, Jackson Laboratory. C57BL/6) were obtained from the Jackson Laboratory and were maintained in the animal facility at Emory University in accordance with institutional guidelines. Male N171-82Q mice were mated with female IL-1RI knockout mice to generate HD-IL1R+/- mice. HD-IL1R-/- mice were then generated by mating HD-IL1RI+/- mice with IL-1RI null mice. PCR genotyping of transgenic mutant htt used the following primers (forward: 5'-CTA CGA GTC CCT CAA GTC CTT CCA GC-3' and reverse: 5'-GAC GCA GCA GCG GCT GTG CCT G-3'). IL-1RI gene deletion was verified by PCR with the following primers (forward: 5'-CCA CAT ATT CTC CAT CAT CTC TGC TGG TA-3' and reverse: 5'-TTT CGA ATC TCA GTT GTC AAG TGT GTC CC-3') for the wild type IL-1RI allele and the primers (forward: 5'-CTG AAT GAA CTG CAG GAC GA-3' and reverse: 5'-ATA CTT TCT CGG CAG GAG CA-3') for the mutated IL-1RI allele. The following PCR condition was used for the wild type and mutant IL1R gene: 94°C for 3 min, followed by 33 cycles of 45 s at 94°C, 45 s at the annealing temperature 63°C, and 60 s at 72°C. For the specific amplification of human htt, the annealing temperature was 64°C. The last cycle was followed by a final elongation step at 72°C for 10 min.

### Antibodies

The anti-huntingtin antibody (rabbit EM48) was previously produced in our laboratory [21]. The mouse anti-gamma-tubulin antibody was purchased from Sigma-Aldrich (St. Louis, MO) and used at 1:50,000 dilution. The mouse antibody to GFAP was obtained from Millipore Inc. Secondary antibodies were peroxidase-conjugated donkey anti-mouse or donkey anti-rabbit IgG (H+L) from Jackson ImmunoResearch (West Grove, PA).

### Rotarod test

Movement coordination performance was evaluated using an AccuRotor rotarod apparatus (AccuScan Instruments). Mice were trained on 2 consecutive days for 3 5-min trials at 5 rpm. Testing was performed on the third day. During testing, the rotating rod was set to accelerate from 0 to 40 rpm in 5 min. Each mouse performed 3 trials on testing day, with 5-min resting periods between each trial. Latency to fall from the rotating rod was measured and averaged for the 3 trials.

### Immunocytochemistry and western blot analysis

Mouse brain regions were carefully dissected from one hemisphere for western blot analysis, and another hemisphere was frozen in O.C.T. on dry ice for cryosectioning. Sagittal sections were cut at 15 μm thickness using a cryostat (Leica CM1850), fixed in 4% paraformaldehyde for 20 min and stained with antibodies as described previously [22]. Mouse EM48 (1:100) and rabbit GFAP (1:1,000) antibodies were used for double immunohistochemical analysis. For DAB (3,3'-Diaminobenzidine) staining, mice were anesthetized and perfused intracardially with phosphate-buffered saline (PBS, pH 7.2) for 30 s followed by 4% paraformaldehyde in 0.1 M phosphate buffer (PB) at pH 7.2. Brains were removed, cryoprotected in 30% sucrose at 4°C, and sectioned at 40 μm using a cryostat. Free-floating sections were preblocked in 3%BSA with 2% normal donkey serum and incubated with rabbit EM48 (1:1,000) as previously described [21]. Western blotting analysis of mouse brain tissues was performed as described previously [21]. Blots were probed with mouse EM48 (1:100), mouse anti-GFAP (1:2,000), and mouse anti-gamma-tubulin (1:50,000).

### Statistical analysis

Results generated from 3 or more independent experiments are expressed as the mean ±SD and were analyzed for statistical significance using a 2-tailed Student's t-test.

## Results

**Figure 1:**
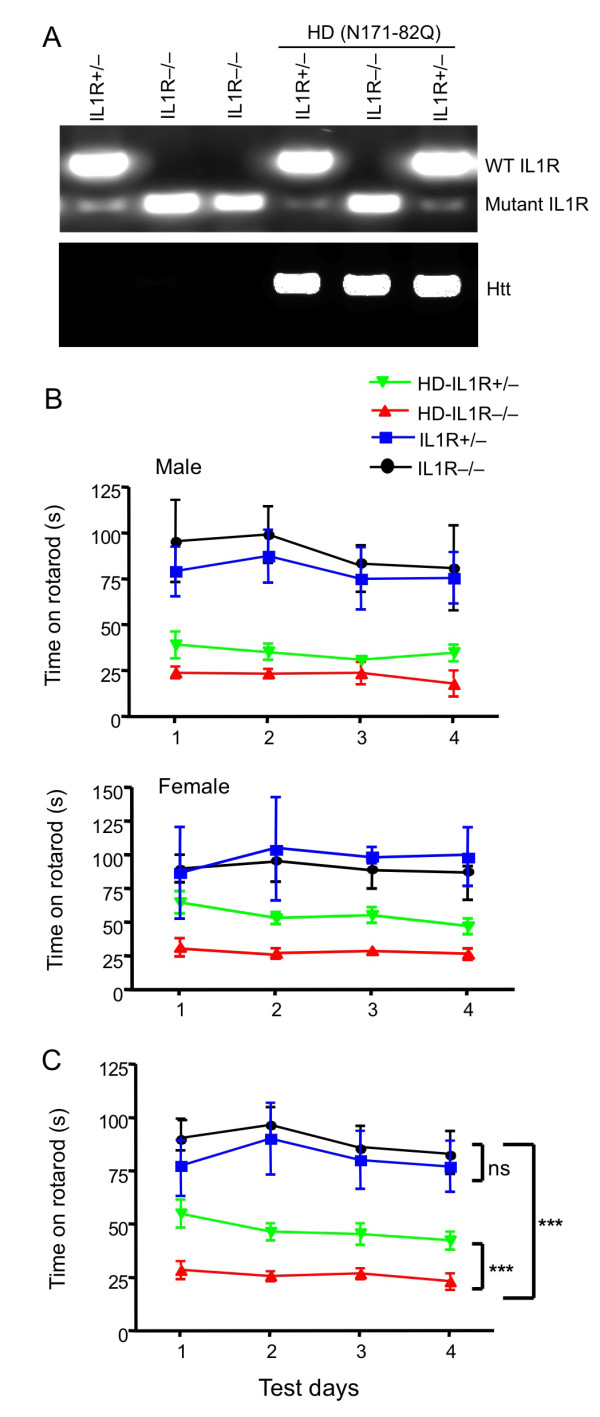
**Lack of IL-1RI leads to rotarod deficits in N171-82Q mice**. **(A) **Genomic DNA PCR analysis of mice of different genotypes. N171-82Q mice with (HD-IL-1R+/-) or without (HD-IL1R-/-) one copy of the IL-1RI gene were used for further comparison. Wild type (350 bp) and mutant (172 bp) IL1R products are indicated. **(B) **Male and female rotarod performances of (HD-IL1R+/-), (HD-IL1R-/-), 1R+/-, and 1R-/- mice at the age of 3 months were examined and compared. n = 10 each group. (C) The rotarod performance of both male and female mice of different genotypes as indicated in (B). n = 10 each group. ** p < 0.01.

There are two homologous receptors for IL-1 (type I IL-1R and type II IL-1R) that can bind either IL-1alpha or IL-1beta. However, type I IL-1R binds IL-1alpha with 10-fold greater affinity than it does IL-1beta. Moreover, type I IL-1, but not type II, receptor is important for IL-1 signaling [12]. Type 1 IL-1R (referred to as IL-1RI) null mice live normally and display no overt phenotypes or behavioral abnormalities [14], allowing us to examine whether a lack of IL-1RI could alter the neurological symptoms of HD mice. To this end, we crossed N171-82Q mice (B6C3F1/J-Tg(HD82Gln)81Dbo/J, *Jackson Laboratory*, Bar Harbour, ME, USA) to IL-1RI knockout mice (B6.129S7-*Il1r1*^*tm1Imx*^/J, Jackson Laboratory. C57BL/6) and generated mice of different genotypes (Figure 1A).

We did not find any significant differences in survival and body weight between N171-82Q mice with and without the IL-1RI gene (data not shown). Rotarod performance has been widely used to assess the motor function of N171-82Q mice [9,21]; we found that N171-82Q mice fell much faster from the rotating rod than IL-1RI knockout mice, indicating a significant motor deficit in HD mice (Figure 1B). There was no significant difference in rotarod performance between female and male IL-1RI knockout mice. Because genetic background might influence mouse behaviors, we focused on the comparison between heterozygous IL-1RI and homozygous IL-1RI knockout mice on the N171-82Q background. These mice carry the same mutations (the mutant htt and targeted IL-1RI genes), so the difference between these two mouse lines is more likely to reflect any effects of IL-1RI expression on HD-related neurological phenotypes. N171-82Q mice lacking IL-1RI (HD-IL1R-/-) performed worse on the rotarod than N171-82Q mice with one copy of the IL-1RI gene (HD-IL1R+/-), regardless of sex (Figure 1B). Combining male and female mice, this difference is statistically significant (Figure 1C), indicating that lack of IL-1RI can exacerbate the motor dysfunction of HD mice.

**Figure 2:**
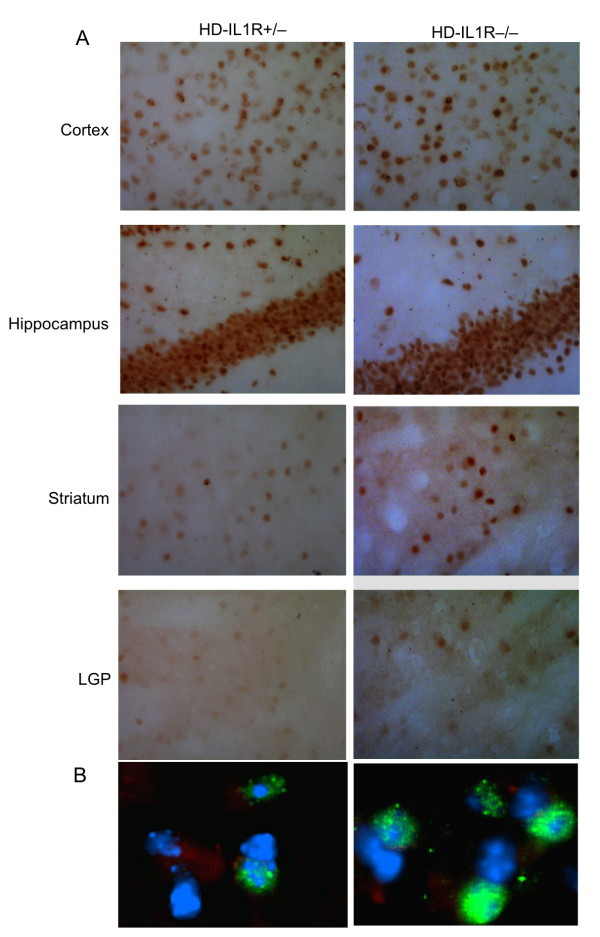
**Increased accumulation of mutant htt in the HD mouse brain when IL-1RI is absent**. **(A) **EM48 immunostaining (20×) showing the increased staining of mutant htt in the brain regions [cortex, striatum, lateral globus pallidus (LGP), hippocampus] of HD-IL1R-/- mice compared with HD-IL1R+/- mice. This increase is more prominent in the striatum of HD-IL1R-/- mice. (**B**) Fluorescent staining showing that mutant htt (green) is more abundant in the nuclei (blue), with small aggregates that are outside the nucleus and that were characterized previously as neuropil htt aggregates.

**Figure 3:**
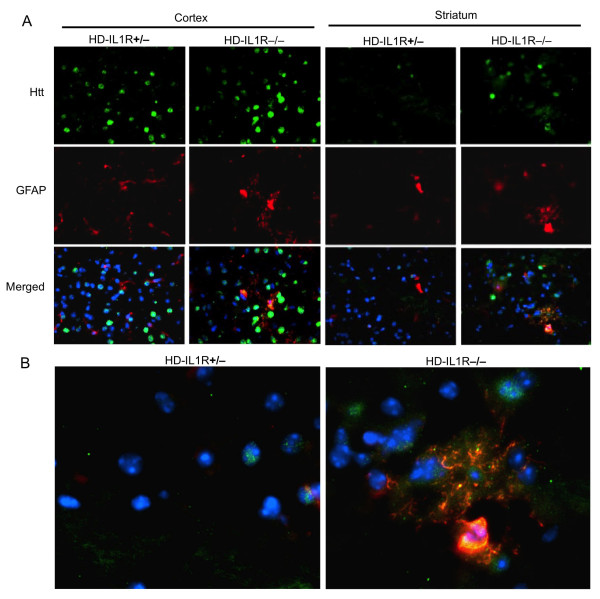
**Increased GFAP staining in the striatum of HD-IL1R-/- mice**. (**A**) EM48 immunofluorescent staining of the brain cortex and striatum of HD-IL1R+/- and HD-IL1R-/- mice showing the selective increase of GFAP staining in the striatum of HD-IL1R-/- mice. (**B**) High-magnification micrographs (63×) showing reactive glial cells with intense GFAP (red) in neuronal cells that also display mutant htt staining (green) in the striatum. The nuclei (blue) were revealed by Hoechst dye staining.

**Figure 4:**
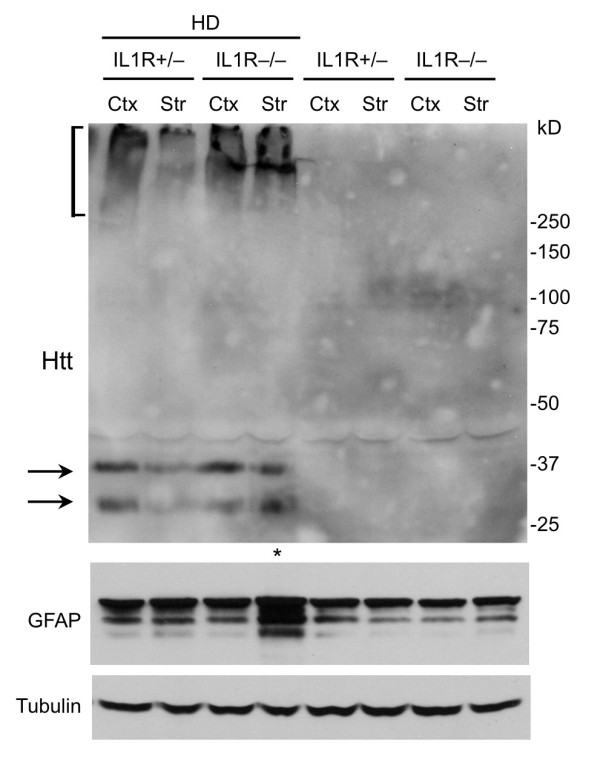
**Western blot analysis of the expression of mutant htt and GFAP in HD mice**. Tissue extracts from the cortex (Ctx) and striatum (Str) of HD-IL1R+/- and HD-IL1R-/- mice were subjected to western blotting with EM48. Aggregated htt is presented in the stacking gel (bracket). Soluble mutant htt is indicated by arrows. Note that htt aggregates and soluble mutant htt are more abundant in the HD-IL1R-/- mouse striatum. The same blot was also probed with antibodies to GFAP and tubulin, revealing an increase of GFAP in the HD-IL1R-/- mouse striatum as well.

We then examined the expression level of mutant htt in different regions of the mouse brain, including the striatum, cortex, hippocampus, and lateral globus pallidus (LGP), a region that is innervated by striatal neurons. Immunohistochemical studies with EM48, an antibody that preferentially reacts with mutant htt [21], revealed that more mutant htt aggregates were present in HD-IL1R-/- mice than HD-IL1R+/- mice (Figure 2A). High-magnification micrographs show that mutant htt formed inclusions as well as small neuropil aggregates in the nucleus (Figure 2B). Increased glial fibrillary acidic protein (GFAP) staining, which reflects early neurodegeneration, has been found in a variety of HD mice [23,24]. Compared with HD-IL1R+/- mice, there were more GFAP-positive cells in HD-IL1R-/- mice in the cortex and striatum (Figure 3A). Increased GFAP staining (red) is clearly shown in high-magnification micrographs that reveal mutant htt (green) in the nuclei (blue) of neuronal cells (Figure 3B). To verify this difference, we performed western blotting analysis of the brain tissues from mice of different genotypes. Htt immunoreactive products that were only present in samples from HD mice represent protein products of transgenic mutant htt (Figure 4). We found that aggregated htt, which is evident in the stacking gel, is more intense in the brain tissue sample from HD-IL1R-/- mice than HD-IL1R+/- mice. This difference is especially pronounced in the striatal tissues. There was also a consistent increase in soluble mutant htt (arrows in Figure 4) in the striatal tissue from HD-IL1R-/- mice. More importantly, the GFAP level was also elevated in the striatal tissue of HD-IL1R-/- mice (Figure 4).

## Discussion

Our findings show that depletion of IL-1RI can exacerbate motor deficits and increase the expression level of mutant htt in the brain, the striatum in particular. Thus, the basal level of IL-1 signaling is likely important for clearing mutant htt in neuronal cells. In N171-82Q mice, N-terminal mutant htt fragments are expressed in neuronal cells, leading to progressive htt aggregation and robust neurological symptoms [9]. The fact that N-terminal mutant htt is prone to protein misfolding and is toxic to neuronal cells is in keeping with the idea that the proteolysis of disease proteins is critical for the pathology of HD and other neurological disorders [8,25-29]. Because these disease proteins progressively accumulate in neurons in HD and other diseases, clearance of these proteins is obviously key for preventing their neurotoxicity. The basal level of IL-1RI signaling may facilitate the clearance of mutant htt.

Although the deleterious role of IL-1 in acute brain injury has been firmly established in the vast majority of experimental models under inflammatory conditions, IL-1 can also regulate neuronal function under normal conditions [30-32]. This regulatory function is found to range from neurotrophic factors-like activity [33] to modulatory action on ion channels [31]. Our studies suggest that the lack of such neuromodulatory functions from IL-1RI can promote the neuronal toxicity of mutant htt. A second possibility is that IL-1 provides neuroprotection under specific experimental conditions (preconditioning), as IL-1 alone given prior to major lesions results in better outcomes, and the administration of IL-1RIa after preconditioning lesions reduces the resistance to injury [34]. The increased accumulation of mutant htt in striatal tissues in the absence of IL-1RI suggests that the basal activity of IL-1RI could regulate the normal function of the ubiquitin proteasome system and autophagy, both of which are important for removing misfolded proteins. Furthermore, the increased GFAP staining in N171-82Q mice [23] suggests that neuronal expression of mutant htt can increase the number of reactive astrocytes. Because IL-1RI in neurons mediates neuronal excitability, whereas IL-1RI in astrocytes may mediate the neuronal protective effects under hostile conditions [35], another possibility is that a lack of IL-1RI in glial cells may attenuate the protective effects of IL-1 on the neuronal toxicity of mutant htt. In most HD mouse models, overt neurodegeneration and apoptosis are not observed. Thus, neuronal dysfunction rather than neuronal loss is more important for neurological symptoms seen in HD mice. Normal glial function is critical for preventing neurodegeneration, and the expression of mutant htt and other misfolded proteins in glial cells can lead to neurological symptoms in transgenic mice [36,37]. Thus, the findings of our study suggest that altering IL-1 signaling as a means of therapy for HD must take into account both its protective and destructive effects.

## Competing interests

The authors declare that they have no competing interests.

## Authors' contributions

The experimental work was designed and performed by CEW. SHL was involved in experiment design and manuscript writing. XJL was involved in experiment design and manuscript writing. All authors read and approved the final manuscript.
